# Multiple Carpometacarpal Dislocations

**DOI:** 10.5811/cpcem.2018.11.41185

**Published:** 2019-01-04

**Authors:** Alexander Davies, Kevin Smith, Wesley Eilbert

**Affiliations:** University of Illinois College of Medicine, Department of Emergency Medicine, Chicago, Illinois

## CASE PRESENTATION

**Image 1:**
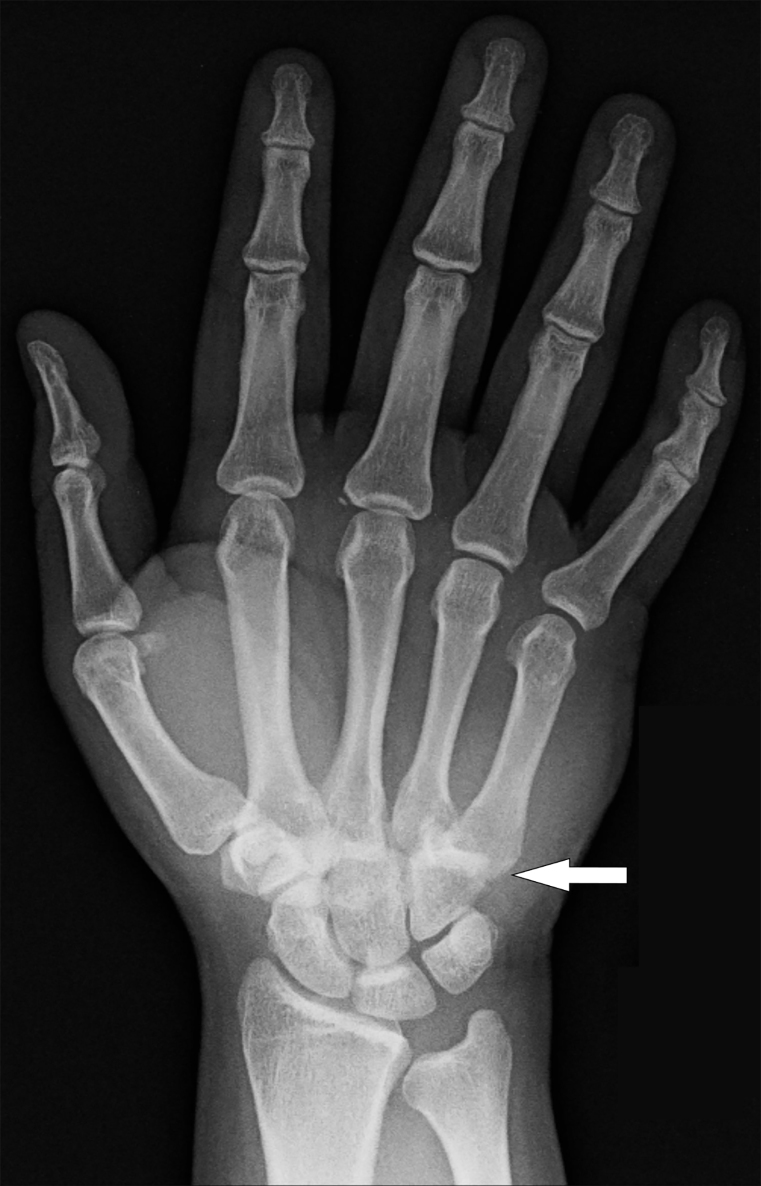
Posterior-anterior view of the right hand showing an obscuring of the second through fifth carpometacarpal joints due to bony overlap (arrow).

**Image 2:**
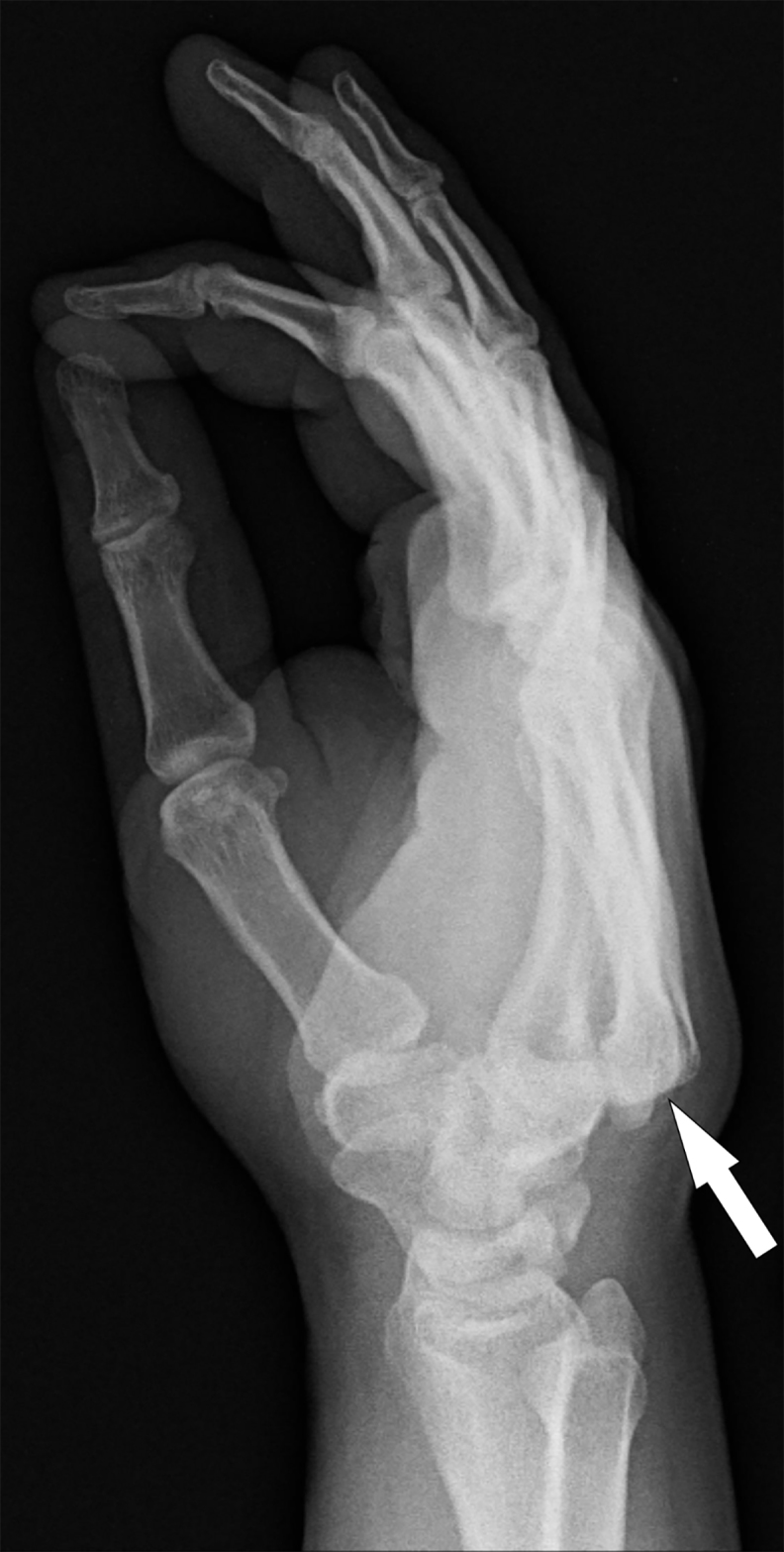
Lateral view of the left wrist showing dorsal displacement of the proximal ends of the second through fifth metacarpals (arrow).

A 30-year-old man presented to the emergency department (ED) complaining of right-hand pain after punching a wall in anger approximately one hour prior to arrival. On examination, there was obvious deformity of the dorsal aspect of the hand with a palpable bony step-off extending across the distal aspect of the wrist. Neurovascular examination of the hand and digits was normal. We obtained standard posterior-anterior (Image 1) and lateral (Image 2) radiographs of the wrist.

## DIAGNOSIS

Carpometacarpal (CMC) dislocations not involving the thumb are rare, accounting for <1% of all hand trauma.^1^ These are typically high-energy injuries occurring in the dominant hand of young men, often as a result of an axial load applied to the metacarpals as occurs with punching.^2^ The diagnosis is frequently missed on initial examination since swelling may obscure the characteristic deformity of the hand dorsum, and routine radiographs may not show the bony displacement clearly.^2^ Left untreated, CMC dislocations frequently result in pain and reduced grip strength.^3^ Most CMC dislocations are dorsal and frequently occur with fractures of the metacarpal base or carpal bones.^4^ Simultaneous dislocation of multiple CMC joints occurs more often than solitary dislocations.^4^

On radiographs, the usual 1–2 millimeter CMC joint space seen on the posterior-anterior view is obliterated by bony overlap, and displacement of the proximal ends of the metacarpals is seen on the lateral view. Closed reduction by applying longitudinal traction to the involved digits with direct pressure over the bases of the dislocated metacarpals should be performed in the ED since delayed reduction is less likely to be successful.^5^,^6^ Operative intervention is indicated if closed reduction is unsuccessful.^5^ This patient had successful closed reduction in the ED using procedural sedation and was discharged with a sugar-tong splint for immobilization.

**CPC-EM Capsule**

What do we already know about this clinical entity?

*Carpometacarpal dislocations not involving the thumb are rare. The diagnosis is frequently missed on initial examination, resulting in significant morbidity*.

What is the major impact of the image(s)?

*It identifies the characteristic obscurement of the carpometacarpal joints on the posterior-anterior view and displacement of the proximal ends of the metacarpals on the lateral view radiographs*.

How might this improve emergency medicine practice?

*Identification of this rare, though commonly missed, injury in the emergency department will prevent long-term hand morbidity*.

## References

[1] Storken G, Bogie R, Jansen EJ (2011). Acute ulnar carpometacarpal dislocations. Can it be treated conservatively? A review of four cases. Hand (NY).

[2] Henderson JJ, Arafa MA (1987). Carpometacarpal dislocation. An easily missed diagnosis. J Bone Joint Surg Br.

[3] Imbriglia JE (1979). Chronic dorsal carpometacarpal dislocation of the index, middle, ring and little fingers: a case report. J Hand Surg.

[4] Fisher MR, Rogers LF, Hendrix RW (1983). Systematic approach to identifying fourth and fifth carpometacarpal joint dislocations. AJR Am J Roentgenol.

[5] Enejat M, de Boer L, Smit JM (2009). A dislocation of the carpal-metacarpal joints of the index, middle and ring finger. Am J Emerg Med.

[6] Kumar A, Olney DB (1994). Multiple carpometacarpal dislocations. J Accid Emerg Med.

